# Diagnostic Value of Diffusion Tensor Imaging for Infants' Brain Development Retardation Caused by Pre-Eclampsia

**DOI:** 10.1155/2021/5545178

**Published:** 2021-07-15

**Authors:** Qing-Na Xing, Yan-Chao Liu, De-Sheng Xuan, Hong-Lei Shang, Xin Zhao, Xiao-An Zhang

**Affiliations:** ^1^Department of Radiology, The Third Affiliated Hospital of Zhengzhou University, Zhengzhou,450052, China; ^2^Institute of Neuroscience, Zhengzhou University, Zhengzhou 450052, China

## Abstract

**Objective:**

Pre-eclampsia (PE) can cause brain development delay in infants. This work aims to characterize the pattern differences of brain white matter development in premature infants under PE conditions and those without.

**Methods:**

Eighty preterm infants delivered by women with PE were selected as the PE group, and ninety-six preterm infants of the same period born to women without high-risk perinatal factors were used as control. All infants underwent diffusion tensor imaging (DTI) examination. The fractional anisotropy (FA) was measured in five regions of interests (ROIs), including posterior limbs of internal capsule (PLIC), splenium of the corpus callosum (SCC), superior frontal gyrus (SFG), superior parietal lobule (SPL), and superior occipital gyrus (SOG). The relationship between the FA values and postmenstrual age (PMA) was analyzed.

**Results:**

After adjusting for the birth weight and gestational ages, in the SCC and PLIC, the PMA and FA values showed a low-to-medium intensity positive correlation in the control group (*r* = 0.30, *p*=0.003; *r* = 0.53, *p* < 0.0001), while no positive relevance was detected in the PE group (*r* = 0.08, *p*=0.47; *r* = 0.19, *p* < 0.08). In the PE and control groups, in the SPL and SOG, the PMA and FA values showed a near-consistent positive correlation (*r* = 0.57, *r* = 0.55 vs. *r* = 0.31, *r* = 0.55; all *p* < 0.05). In the control group, in SFG, the PMA and FA values had a medium intensity positive correlation (*r* = 0.47, *p* < 0.0001), but there was no statistical difference in correlation in PE (*r* = 0.10, *p*=0.39).

**Conclusion:**

PE may cause lagging brain development in the SCC, PLIC, and SFG during infancy. DTI may be an effective and sensitive detection tool.

## 1. Introduction

The incidence of hypertension disorder complicating pregnancy is increasing, corresponding with increases in maternal, fetal, and neonatal morbidity and mortality [1]. Pre-eclampsia (PE) is among the most prominent of complications. In recent years, there have been multiple breakthroughs in elucidating the mechanism of maternal and child injury caused by PE [2]. PE can lead to neurodevelopmental impairments (NDIs) in offsprings [3–5]. Therefore, it is necessary to find a sensitive tool to predict and detect delayed brain development in infants caused by PE.

To date, computed tomography, ultrasound, and conventional magnetic resonance imaging (MRI) make it possible to estimate the microstructure of the infants' brains. Studies have concluded that structural abnormalities of cranial MRI in infancy and childhood predict poor long-term neurological prognosis [6–8]. However, this approach has limitations when estimating white matter brain development quantitatively.

Subtle white matter (WM) damage in the brain can be easily detected by diffusion tensor imaging (DTI), which is a noninvasive technique [9, 10]. Fractional anisotropy (FA) is one of the important parameters of DTI. The FA values reflect the proportion of the water molecule anisotropy component in the whole diffusion tensor and indirectly reflect the integrity of the white matter fiber bundle [11]. Demyelination and axonal alterations, and WM fibers damage, may result in changes in the diffusion of water molecules and therefore lead to a change in the FA values [12]. In the recent literature, abnormal FA values of brain WM in the offspring of mothers with gestational diabetes mellitus were significantly related to worse neurocognitive performance [13]. The FA value has good indicative value for long-term neurological prognosis [14, 15]. To our knowledge, DTI (FA values) has not been used to evaluate brain development in infants of mothers with PE.

In this work, we aimed to explore whether patterns of brain development assessed by DTI differ between PE group infants and matched typical children.

## 2. Materials and Methods

This study was approved by the ethics Committee of the hospital ((2020) medical ethics review no. 30), and the informed consent and signature of the guardians were obtained. A retrospective analysis of clinical and imaging data of 176 premature infants in the Imaging Department of our Hospital from July 2015 to July 2019 was performed. Eighty premature infants born to women with PE were included as the PE group, including 47 males and 33 females. The gestational age was 27^+6^∼36^+6^, with an average of 30^+5^ weeks. All mothers' blood pressure was well-controlled during pregnancy. In the same period, ninety-six premature infants delivered by women without high-risk perinatal factors were selected as the control group, including 51 males and 45 females. The gestational age was 25^+6^∼36^+5^, with an average of 30^+1^ weeks.

### 2.1. Diagnostic Criteria of PE

PE was initially defined as a rise in the systolic blood pressure to ≥140 mmHg or in diastolic pressure to ≥90 mmHg on two separate occasions in a patient who was previously normotensive, as well as a proteinuria level of ≥300 mg in a 24-hour collection, of 0.3 g/g by urine protein : creatinine ratio or +1 by urine dipstick if quantitative methods are unavailable, occurring after 20 weeks of pregnancy. The updated classification eliminated proteinuria as a requirement for diagnosis in the presence of other end-organ damages, such as thrombocytopenia, impaired liver function, new renal insufficiency, pulmonary edema, or new-onset cerebral or visual disturbances [16].

### 2.2. Exclusion Criteria

1. Pregnant women with hematological disorders, a history of chronic pulmonary disease, general problems such as kidney or heart disease, metabolic diseases such as hyperthyroidism, nutritional problems such as hyperemesis gravidarum, mothers with uterine and placental abnormalities, and a history of smoking, with twin-twin transfusion syndrome during pregnancy were excluded
2. Premature infants who were born after 37 weeks of gestation, with chromosomal, genetic, or structural defects and severe brain injury/encephalomalacia and if their clinical condition was too unstable to be suitable for magnetic resonance imaging were excluded

### 2.3. Data Collected from Pregnant Women and Newborns

1. Pregnant women: demographic data, obstetric history, data on hypertensive disorders of pregnancy, and birth outcomes were documented from the case notes, as well as a structured questionnaire. Trained research nurses blinded to the MR imaging findings reviewed medical records and extracted clinical data.
2. Premature infants: information regarding sex, gestational age, birth weight, Apgar scores, and types of congenital heart disease amalgamated at birth were collected.

### 2.4. Image Data Collection

Brain MRI studies were performed with a Magnetom Skyra 3T MRI clinical scanner (Siemens, Erlangen, Germany). Each infant was injected with 5 mg/kg phenobarbital before falling asleep. The cotton balls were placed in the external auditory canal to reduce the effect of equipment noise, and sponges were fixed on both sides of the head to reduce motion artifacts. During the DTI scans, the patients' condition was stable and in continuous intensive care of a doctor in the Neonatal Care Center. An experienced radiologist in MRI reported all the images. All images met the diagnostic requirements.

Acquisition was achieved with a head 20 coil, with the following sequences:

1. Sagittal T1WI-MPRAGE scanning parameters: number of sections, 192; field of view (FOV), 240 mm × 240 mm; section thickness, 0.9 mm; repetition time (TR), 2000 milliseconds; echo time (TE), 2.32 milliseconds; flip angle, 8 degrees; resolution, 256 × 256; acquisition times, 1; and scanning time, 4 minutes 40 seconds
2. Ep2d_diff_mddw scanning parameters: number of sections, 25; field of view (FOV), 200 mm × 200 mm; section thickness, 4.0 mm; repetition time (TR), 3700 milliseconds; echo time (TE), 92 milliseconds; *b* values, 0, 1000 s/mm^2^; resolution, 128 × 128; number of excitation, 1; the direction of diffusion sensitive gradient field, 20; and scanning time, 4 minutes 39 seconds

### 2.5. Measurement of FA

**Figure 1:**
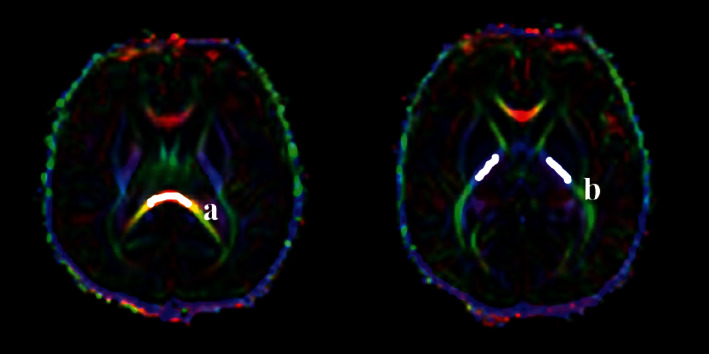
Color FA maps in the infantile brain. Examples of ROIs for DTI are shown in white color. Regions of interest: (a) splenium of corpus callosum; (b) posterior limb of internal capsule.

**Figure 2:**
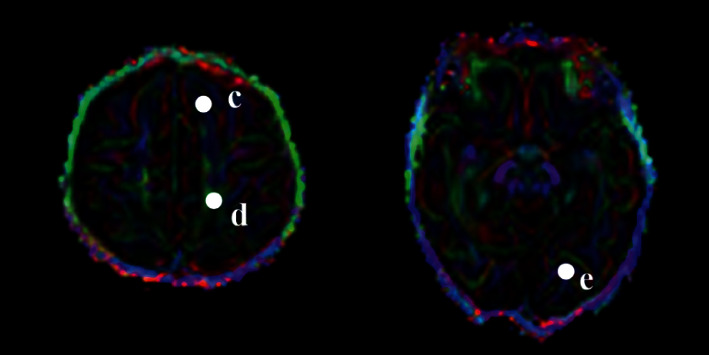
Color FA maps in the infantile brain. Examples of ROIs for DTI are shown in white color. Regions of interest: (c) superior frontal gyrus; (d) superior parietal lobule; (e) superior occipital gyrus.

All images were automatically entered into a postprocessing workstation (Siemens syngo.via) in DICOM format to generate an FA map. The size of the ROI was controlled at 10 ± 2 mm^2^. Five ROIs (Figures 1 and 2) were measured, including posterior limbs of internal capsule (PLIC), splenium of the corpus callosum (SCC), superior frontal gyrus (SFG), superior parietal lobule (SPL), and superior occipital gyrus (SOG). All ROIs were placed in the center of the anatomical position, and the average bilateral FA values were taken after measuring the symmetrical parts of the bilateral cerebral hemispheres. The FA values of each ROI were measured three times before taking the average value. The FA map was analyzed by two experienced doctors with senior professional titles in the imaging department, and two researchers blind to the clinical data performed the measurements separately. If there was a disagreement in the measurement process, it was resolved through consultation.

### 2.6. Statistical Analysis

Data were analyzed using SPSS for Windows, version 25 software (SPSS Inc., Chicago, IL, USA) and GraphPad Prism 8.0.2 (GraphPad Software Inc.). The normality of measurement data was assessed using the Shapiro–Wilk test. The data are expressed as the mean ± standard deviation ($\bar{x}\pm s$). Normally distributed variables were compared using Student's *t*-test of variance, whereas nonnormally distributed variables were compared using the Mann–Whitney *U*-test. Univariate analyses of categorical data were performed using Fisher's exact chi-square test or the chi-square test.

Correlation analysis was conducted between FA values and postmenstrual age after adjusting other factors (birth weight and gestational weeks). Firstly, residuals from the regression of these FA values and postmenstrual age on these confounding factors were calculated. Secondly, we added to these residuals the corresponding mean FA values and mean postmenstrual age and plotted these adjusted clinical variables against the adjusted FA values. And, the formula was *Y* = *b*0 + *b*1 ∗ *X*. *p* < 0.05 is considered statistically significant.

## 3. Results

### 3.1. Baseline between the Control and PE Group

Compared to the control group, preterm babies in the PE group had a slightly larger gestational age and lower birth weight (all *p* < 0.05). The remaining other factors did not show statistical significance between the two groups (all *p* > 0.05) (Table 1).

### 3.2. Comparison of Brain Development between Two Groups (after Adjusting for Birth Weight and Gestational Ages)

**Figure 3:**
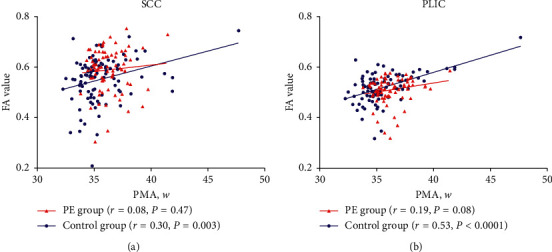
Patterns of brain development in the SCC and PLIC regions of the two groups during infancy (after adjusting for birth weight and gestational ages).

**Figure 4:**
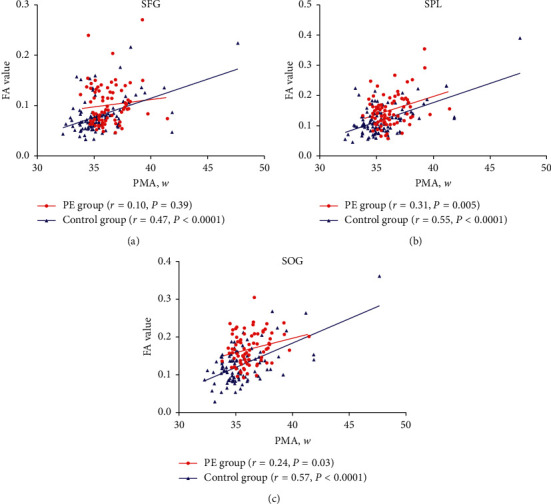
Patterns of brain development in the SFG, SPL, and SOG regions of the two groups during infancy (after adjusting for birth weight and gestational ages).

1. Compared to control group, the slope of the formula is smaller in the PE group (Table 2).
2. In the SCC and PLIC area, the PMA and FA values showed a low-to-medium intensity positive correlation in the control group (*r* = 0.30, *p*=0.003; *r* = 0.53, *p* < 0.0001). The PMA and FA values showed a positive relevance in PE group, but the correlation was not statistically significant (*r* = 0.08, *p*=0.47; *r* = 0.19, *p*=0.08) (Figure 3).
3. In the SPL and SOG of the PE and control groups, PMA and FA values had a nearly consistent positive correlation (*r* = 0.31, *p*=0.005; *r* = 0.55, *p* < 0.03 VS *r* = 0.57, *p* < 0.0001; *r* = 0.55, *p* < 0.0001). In the SFG area of the control group, the PMA and FA values had a medium intensity positive correlation (*r* = 0.47, *p* < 0.0001), while no statistical significant in positive correlation was found in the PE group (*r* = 0.10, *p*=0.39) (Figure 4).

## 4. Discussion

An initial objective of the study was to make further understanding of the diagnostic value of diffusion tensor imaging for infants' brain development retardation caused by PE.

The results of this study mainly showed three points: (1) possible accounts of NDI caused by PE. (2) PE may cause poor brain development in the SCC, PLIC, and SFG regions. (3) No backward brain development was found in the in the SPL and SOG regions in the PE group.

Some factors may be closely related to brain development. Gestational age should be considered first. Previous studies have concluded that early preterm birth is associated with a high level of attention-deficit/hyperactivity disorder (ADHD) symptoms in preschool children [17]. Gestational age is dimensionally related to structural brain network abnormalities across development [18]. In addition, lower birth weight may cause lagging brain development [19]. Previous studies found brain FA values vary among brain ages [20]. Therefore, in order to explore the correlation between FA values and PMA in both groups, the gestational age and birth weight should be adjusted.

Possible accounts of NDI caused by PE: in this study, the PE group had a higher birth probability of SGA. One study has found differences in brain development between SGA and normal control fetuses [21]. And, some scholars hold that PE may contribute to the birth rate of SGA and that it is closely related to placental function [22]. PE is believed to be a severe manifestation of placental dysfunction due to early angiogenic imbalances and inflammatory disturbances [23]. Studies have found that the source of damage to mother and child caused by PE is the placenta [5]. Placenta vascularization seems to be common features in pre-eclampsia, which could lead to intrauterine growth restriction (IUGR) mainly due to reduced placental blood flow and chronic hypoxia [24]. In clinical practice, PE may increase the possibility of IUGR. Previous work has suggested that brain network efficiencies in children with IUGR are decreased, and children with IUGR have a higher risk of inattention/hyperactivity and/or dysfunction, and the decrease in brain network efficiency is related to the decrease in FA weighted connectors [25]. The above suggests that PE may cause IUGR and increase the risk of SGA birth, which may be associated with closely related neonatal neurological developmental defects. It may also indicate that alterations in placental structure and function are associated with the brain development in offspring.

In two groups, the FA values continued to increase with the increase of PMA in all white matter regions during infancy. This is related to the myelination of the fiber tracts, which is consistent with previous findings [9, 26, 27]. This work revealed the generally consistent pattern of brain development in two groups. Thus, the poor neurological prognosis caused by PE may be due to lagging brain development.

The correlation between PMA and FA values (SCC and PLIC) was lower in the PE group than in the control group. Previous study finds FA values changes with brain age [20]. Thus, PE may lead brain development lag in the SCC and PLIC regions. Some scholars found subjects with autistic spectrum disorder (ASD) exhibited reduced FA values in corpus callosum and PLIC [10, 28]. Some studies also suggested that hypertensive disorders complicating pregnancy increase the risk of attention-deficit/hyperactivity disorder (ADHD) [29, 30]. The above suggests that DTI examination may be a sensitive tool in monitoring the risk of potential brain development lag caused by PE and might provide a better assessment of the risk of developing ASD or ADHD.

PE may cause lagging brain development in the SFG regions, but no backward brain development was found in the in the SPL and SOG regions. Some studies have shown basic patterns of brain maturation processes, for example, FA values increases with age [20]. Maturation direction is from posterior to anterior and center to peripheral, which is similar to the maturation pattern of the frontal lobe to occipital lobe, and the FA values of the central brain tissue had the highest increase [26]. An earlier increase in FA values indicates earlier myelin formation and mature structural connectivity formation in these regions. The SPL and SOG regions are the first to myelin formation, suggesting that the degree of white matter myelination in these two regions was already more consistent in both groups at the DTI examination or that the PE group may have caught up with the control group. According to this brain maturation pattern, PE may insult brain development in the SFG during infancy. However, we should select multiple ROIs for analysis, increase sample size, and further expand the time span of PMA in the future. The above illustrates that DTI examination may be useful in detecting backward brain development caused by PE and providing evidence for early intervention.

Some limitations of our study should be mentioned. Firstly, risk factors such as maternal BMI and metabolic factors for early pregnancy were not included, which are unknown because these data are not available. Secondly, there is unavoidable subjectivity when using an ROI method. Thus, we removed clinical information of the patients before measuring, so the average value on the left and right side was acceptable. We adjusted the size of ROI by each anatomical structure. Thirdly, we used cross-sectional data in this study, as obtaining longitudinal data in the same infant is difficult. We will continue to follow-up the infants included in this study.

## 5. Conclusion

In this work, we detected significant microstructural WM development retardation in particular regions (SCC, PLIC, and SFG) in PE-exposed infants when compared with unexposed controls. This might reveal that PE directly insults the brain development of the offspring. This might reveal that DTI examination shortly after birth may be helpful in detecting brain development lag behind caused by PE. It is valuable for pediatricians to discover the early abnormality of the individuals and then initiate proper and early clinical intervention.

## Figures and Tables

**Table 1:** Baseline of the control group vs. PE group ($\bar{x}\pm s$).

Characteristic	PE group (*n* = 80)	Control group (*n* = 96)	*p* value
*Maternal*			
Maternal age, years	31.11 ± 5.02	31.91 ± 4.41	0.146
*History of gestation*			
Pregnancy times	1.79 ± 1.62	1.73 ± 1.38	0.942
Breeding times	0.79 ± 0.87	0.78 ± 0.88	0.841
Number of miscarriages	0.98 ± 1.33	0.95 ± 1.03	0.690
Antenatal corticosteroids, *n*	64	71	0.345
*Neonatal*			
Gestational age, weeks	30.79 ± 1.79	30.10 ± 2.02	0.005^*∗*^
Birth weight, g	1287.81 ± 398.98	1395.95 ± 320.64	0.002^*∗*^
*Mode of delivery, n*			
Spontaneous vertex	4	3	0.350
Caesarean section	76	93	0.703
Boy/girl	47/33	51/45	0.454
One of the twins	14	26	0.131
1 min apgar	7.81 ± 1.54	7.71 ± 1.62	0.716
5 min apgar	8.63 ± 1.41	8.56 ± 1.26	0.579
*Neonatal complications at birth, n*			
Atrial septal defect	50	67	0.308
Ventricular septal defect	1	0	0.455
Patent ductus arteriosus	31	44	0.114
Patent foramen ovale	25	18	0.055
Pulmonary hypertension	13	14	0.764
Number of congenital heart disease	1.48 ± 0.31	1.45 ± 0.93	0.945
Hyperbilirubinemia	74	89	0.958
NRDS	77	93	1
SGA	14	5	0.009^*∗*^
BPD	17	27	0.294
NEC	3	3	1
PMA at DTI examination, *w*	36.24 ± 1.43	35.56 ± 2.62	0.008^*∗*^
PNA at DTI examination, *d*	38.15 ± 13.16	38.04 ± 17.44	0.477

The mean ± standard deviation (SD) was reported; ^*∗*^*p* < 0.05. *p* values were obtained by using Fisher's exact chi-square test, chi-square test, Student's *t*-test and Mann–Whitney *U*-test. Abbreviations: PMA: postmenstrual age; PNA: postnatal age; NRDS: neonatal respiratory distress syndrome; SGA: small for gestational age; BPD: bronchopulmonary dysplasia; NEC: necrotizing enterocolitis.

**Table 2:** The parameters of the linear regression formula between PMA and FA values in two groups.

	*R* ^2^	95CI%	Formula
SCC-PE	0.007	−0.140∼0.297	*Y* = 0.005 ∗ *X* + 0.406
SCC-control	0.088	0.102∼0.469	*Y* = 0.012 ∗ *X* + 0.130
PLIC-PE	0.037	−0.030∼0.396	*Y* = 0.007 ∗ *X* + 0.248
PLIC-control	0.277	0.364∼0.656	*Y* = 0.014 ∗ *X* + 0.039
SFG-PE	0.009	−0.125∼0.310	*Y* = 0.003 ∗ *X* − 0.004
SFG-control	0.277	0.364∼0.657	*Y* = 0.008 ∗ *X* − 0.188
SPL-PE	0.097	0.097∼0.496	*Y* = 0.012 ∗ *X* − 0.281
SPL-control	0.221	0.297∼0.613	*Y* = 0.013 ∗ *X* − 0.331
SOG-PE	0.096	0.097∼0.496	*Y* = 0.008 ∗ *X* − 0.107
SOG-control	0.059	0.0236∼0.439	*Y* = 0.013 ∗ *X* − 0.331

## Data Availability

The data used to support the findings of this study are available from the corresponding author upon request.
